# Fiberfill©—A New Bone Substitute for Treatment of Chronic Osteomyelitis?

**DOI:** 10.3390/jcm15031277

**Published:** 2026-02-05

**Authors:** Hendrik Schöllmann, Veronika Weichert, Claas Neidlein, Nikolaus Brinkmann, Marcel Dudda, Eva Steinhausen

**Affiliations:** 1 Department of Orthopedic and Trauma Surgery, BG Klinikum Duisburg, Großenbaumer Allee 250, 47249 Duisburg, Germany; 2 Department for Arthroscopic Surgery, Sports Traumatology & Sports Medicine, BG Klinikum Duisburg, Großenbaumer Allee 250, 47249 Duisburg, Germany; 3 Department of Trauma, Hand and Reconstructive Surgery, University Hospital Essen, University of Duisburg-Essen, Hufelandstraße 55, 45147 Essen, Germany

**Keywords:** osteomyelitis, Fiberfill©, bone reconstruction, fracture related infection, bone substitute, bone healing

## Abstract

**Background/Objectives**: Therapy of osteomyelitis first aims to control infection of the bone and surrounding tissue. Once that is achieved, surgeons are regularly faced with defects of the bone as a result of the infection. Reconstruction of the bone is necessary. In the past years, various bone substitutes were developed. Since 2022, we have used Fiberfill© as a new allogenic material. The aim of this study is to analyze the outcome of patients with chronic osteomyelitis who received bone reconstruction with Fiberfill©. **Methods**: Patients who suffered from chronic osteomyelitis and received Fiberfill© for bone reconstruction between October 2022 and July 2024 were retrospectively analyzed. Endpoints were infection control, bone healing and function in terms of weight bearing. Data was analyzed descriptively. **Results**: 38 patients with a mean age of 55.6 years ± 16.4 years standard deviation were analyzed and seen for clinical and radiographic control after surgery with a mean follow up of 60 weeks up to three years. Mean defect size was 2.4 cm. Thirty-four patients (89%) did not have any re-infection. Complications associated with Fiberfill© were not found in any patients. Healing of the bone (completely and partially) was found in 35 patients (92%). Twenty-nine patients (78%) walked fully weight bearing. Seven patients were active with partial weight bearing at time of last follow-up (19%). **Conclusions**: Fiberfill© can be regarded as a bone substitute for reconstruction of bone defects in therapy of osteomyelitis. We did not find clear disadvantages or a high number of complications after filling up bone defects with Fiberfill©.

## 1. Introduction

Chronic osteomyelitis (COM) represents a major complication in orthopedic and trauma surgery [1]. The inflammatory bone condition is characterized by necrotic bone and affected surrounding tissue. During hematogenous infection, causative pathogens of COM are seeded into the bone secondary to a bloodstream infection. This condition occurs mostly in geriatric patients and immuno-compromised populations. Non-hematogenous infection of bone occurs after direct exposure to pathogens, e.g., from open fractures in trauma or surgery setting [2]. Prevalence of COM continues to rise, particularly among elderly patients [3]. The treatment of COM consists of surgical and antibiotic therapy [4]. Gaining control of the infection is the primary goal [2]. Avascular necrosis of bone and formation of sequestrum necessitate surgical debridement [5]. During surgery, tissue samples are extracted from the site of infection and passed on for being microbiologically analyzed. The causative pathogen can be identified, thus allowing more precise antibiotic therapy [6].

Formation of biofilm, which consists of polysaccharides, proteins and extracellular DNA, protects bacteria within it. The biofilm on necrotic bone limits antibiotic diffusion to bacterial cells and inhibits immune cell penetration [7]. Biofilm can be formed by bacteria such as *Staphylococcus*, *Streptococcus* and *Pseudomonas* [8]. In addition, species such as, e.g., *Staphylococcus*, have the ability of abscess formation in chronic infection sides. Abscess formation results in restricted blood flow in that area, which can increase the severity of infection [9]. Low antibiotic concentrations at the site of infection due to impaired blood supply and drug-resistant pathogens can cause a protracted healing process [10,11]. These mechanisms can result in the necessity for several surgical debridements.

Surgical therapy for infection control, as well as the infection itself, cause defects of the bone and tissue. After control of the infection is achieved, bone reconstruction is necessary.

The gold standard is the autologous bone graft, which has osteoinductive, osteogenic and osteoconductive properties [12]. However, there are various disadvantages which come along with autologous bone grafts. Prolonged surgery time can be demanding for patients and physicians and potentially dangerous for those with underlying health condition. Depending on the size of defects, the restriction of volume of autologous bone grafts can make bone substitutes necessary. Donor site morbidity is considerable, and accounts up to 14% with major donor-site complication rates of 2.4 to 6.2% [13,14]. These disadvantages of autologous bone graft have resulted in increasing use of allogenic bone substrates over recent years. New substrates are compared to the well-established practice of bone reconstruction with autologous grafts [15].

During recent years, several bone substitutes have been developed. Bone substitutes can be subdivided into allogenic processed bone grafts and synthesized bone grafts. Within allogenic bone grafts, demineralized bone matrix (DBM) retains some of its osteo-inductive properties during chemical processes and sterilization. While the osteoinductive properties are an advantage, the mechanical stability of DBM is rather poor [16]. Within the group of synthesized bone grafts, polymers and calcium-based materials provide osteoconductive properties and feature the advantage of mechanical stability, which can be useful in larger defects [17]. Some materials, e.g., ceramics, can be enriched with antibiotics [18]. Others, such as bioactive glass, offer antimicrobial features due to their structure. For bone reconstruction in COM, bone substitutes offering antibacterial properties are preferably used [15].

Since 2022, Fiberfill© has been used in our institute as an allogenic processed bone graft for bone reconstruction. The allogenic bone graft consists of demineralized bone matrix (75%) and freeze-dried cancellous bone (25%) [19]. The clinical alteration of Fiberfill© in comparison to other grafts is, especially, the moldability, which it regains after dilution in liquid. After molding into the bone defect, due to its viscoplastic characteristic, Fiberfill©, in large part, stays in place. For supplying mechanical stability short term, it is certainly not eligible.

To our knowledge, there is no published data about bone reconstruction with Fiberfill© in the treatment of chronic osteomyelitis.

The aim of this study is to follow up and analyze the course of osteomyelitis patients who received Fiberfill© for bone reconstruction in our Level-I-Trauma Center. We hypothesize that Fiberfill© used for reconstruction of bone defects in COM is adequate.

## 2. Materials and Methods

### 2.1. Study Design and Patient Cohort

In this retrospective single-center study, surgically treated patients who received Fiberfill© for bone reconstructions in COM were included. Inclusion period in our level I Trauma center in Germany was between 1 October 2022 and 30 July 2024, regarding surgical bone reconstruction.

Inclusion criteria comprised chronic osteomyelitis or infected non-union (positive bacteriology or positive histology) and bone reconstruction of a defect using Fiberfill© as an allogenic bone graft. Follow up was recorded until 1 October 2025, at the latest.

Exclusion criteria were open growth plates in radiographic imaging, non-compliance (with weight bearing and prescribed antibiotic therapy), attendance at less than one follow-up consultation, and aseptic non-unions.

The primary endpoint was full bone healing. Secondary endpoints of the study were absence of re-infection and weight-bearing capacity.

Identification of patients and further information was obtained from the institutional databank.

Information retained from the hospital computer system was divided into information prior to bone reconstruction, data collected during index surgery (bone reconstruction), and data collected during time of follow-up.

Pre-surgery information included: epidemiologic data, fracture-specific information, type of bacteria, size of bone defect (measured in radiographic exam before reconstructive surgery); number of surgeries undertaken before reconstruction; and restitution-limiting diseases/medication. See also Table 1.

Peri-operative data contained, e.g., bacteriology (in index surgery); change of pathogen; volume of embedded Fiberfill©; the combination of Fiberfill© with other bone substitutes/bone graft; and information about osteosynthesis and antibiotic therapy.

Additional information regarding the follow-up was registered, such as last follow-up visit; bone healing; weight bearing, and re-infection. Concerning the attribution partial-/full- bone healing, evaluation was done by X-ray or CT-imaging. Complete bone healing was defined as the healing of at least three out of four cortices or no evidence of the fracture line. Partial bone healing was defined as an increase of bone-fusion in comparison to the last imaging, while no complete bone healing is identified.

All the above-mentioned information was processed anonymously.

### 2.2. Statistical Analysis

Data was transferred to and collected in Microsoft Excel (Microsoft 365 MSO, Version 2410, Microsoft Corp., Redmond, WA, USA) and analyzed descriptively. The analysis included calculation of the mean, the standard deviation (SD) and time differences for different dates. Furthermore different subgroup analyses were performed, comparing bone healing to time of follow-up and bone-healing to defect size.

### 2.3. Fiberfill©

Fiberfill© was licensed in 2021 by “Deutsches Institut für Zell- und Gewebeersatz” in Berlin, Germany, and consists of demineralized bone matrix (75%) and freeze-dried cancellous bone (25%) [19]. After hydration, this bone substitute can be formed and precisely fitted into the defects. Dilution with antibiotics is possible, to reach a higher local concentration of antimicrobials.

### 2.4. Surgery and Clinical Treatment

All patients received treatment through the department of septic surgery, including surgical debridements and bone reconstruction. In all cases, radical debridement was carried out prior to bone reconstruction and implantation of the allogenic bone graft.

Antibiotic treatment was chosen according to the bacteriology and the antibiotic resistance pattern from prior surgical debridements. Standard antibiotic treatment according to the resistance pattern included a cephalosporin. Perioperatively, the treatment was carried out intravenously. Bone reconstruction was performed only after clinical control of the infection. Decision on infection control was made by the team of septic surgery following the definition given by Metsemakers et al. Confirmatory criteria are fistula, sinus, and wound breakdowns, as well as purulent drainage. Suggestive criteria include local signs such as redness, increased local temperature, and fever (≥38 °C). Radiographic signs such as bone lysis, implant loosening, sequestration, and laboratory results (white blood cell count, C-reactive protein) were considered. Intraoperatively, identification of two pathogens in culture was defined as confirmatory presence of infection [20]. Absence of these criteria was considered as “infection control”.

During bone reconstruction, tissue samples for bacteriology were taken from all patients. Duration of continued antibiotic treatment was decided after antibiotic stewardship consultation in accordance with the institute of microbiology for each patient individually.

### 2.5. Ethical Approval

For this retrospective single-center study, the ethical committee of medical association Nordrhein (“Ärztekammer Nordrhein”) was consulted for approval. Due to the study design, no ethical inquiry was necessary.

## 3. Results

Thirty-eight patients received bone reconstruction with Fiberfill© after infection control of osteomyelitis in our institute between October 2022 and July 2024. Fourteen patients were female, twenty-four male (37% vs. 63%). The average age of the collective was 55 years ± 16.4 (SD).

### 3.1. Preoperative Results

Three patients of our collective were prescribed, and in need of, immunosuppressive medication. Five patients suffered from restitution-limiting co-morbidities (diabetes, peripheral artery disease). The majority of patients (82%) did not require immunosuppression nor suffer restitution-limiting diseases.

Six patients suffered from osteomyelitis of the upper limb (16%). Most chronic infections were located in the lower limb (femur, tibia and fibula), with a total of 28 patients (74%). In three cases (8%), the foot was affected. One patient suffered from COM of the pelvis.

The ratio of diaphyseal and metaphyseal defects was approximately balanced with 53% for metaphyseal and 47% for diaphyseal localization.

Thirty-two percent of the infections were a consequence of open fractures; 68% of our collective did not primarily have open fractures.

**Figure 1:**
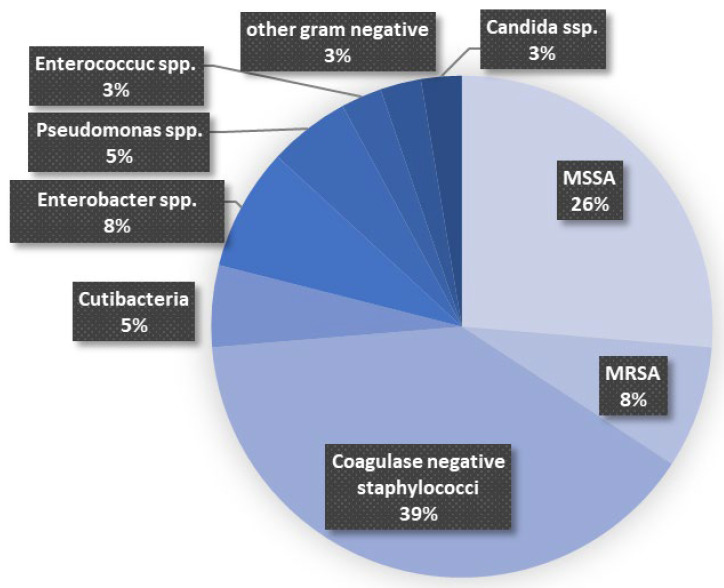
Pathogen findings before reconstructive surgery.

The most common pathogen involved was coagulase negative *Staphylococci* (39%), followed by methicillin-sensitive *Staphylococcus aureus* (MSSA) (26%). Further bacteria detected before reconstructive surgery are displayed in Figure 1.

Bone reconstruction was performed after a mean of 19 ± 22 weeks (SD) after diagnosis of infection. In one patient, reconstruction was performed when, for the first time, a pathogen had been detected (coagulase negative *Staphylococci*).

The size of bone defects measured in radiography varied from 0.5 to 7.5 cm, with a mean of 2.4 cm. Standard deviation was calculated to ±1.9 cm.

Before bone reconstruction, sixteen patients (42%) received more than four, and up to nine, surgeries. In five cases (13%), ten or more surgeries had been performed already.

### 3.2. Intraoperative Results

In thirteen patients (34%), pathogens were found in the tissue samples which were taken during bone reconstructive surgery. In 66%, no bacteria were found intraoperatively. Of those cases with intraoperative findings during bone reconstruction, in 62% the pathogen did not change respectively to prior identified and treated species.

The volume of Fiberfill© used to fill up the defect for bone reconstruction varied between 5 and 10 cubic centimeters (ccm) and reached up to 15 ccm in one case. The mean value of the implanted volume was calculated at 8 ccm ± 3 ccm (SD).

In seventeen surgeries (45%), the allogenic bone substitute was combined with either another bone supplement, autologous bone graft, or bone morphologic protein. From these seventeen combined surgeries, nine surgeries (53%) were performed combining autologous bone graft with Fiberfill©. Bioactive glass (S53P4) was used four times (24% of combined surgeries, 11% overall) and bone morphologic protein II was used five times (29% of combined surgeries, 13% overall).

Twenty-one patients received Fiberfill© as a solitary bone substitute (55%).

The ratio of patients with maintained osteosynthesis and those with removal of existing fixation and re-osteosynthesis was 42% to 58%, so that the majority in our collective did receive a renewal of the osteosynthesis.

In addition to the intravenous perioperative therapy, 11% (four patients) of our collective received local antibiotic therapy (vancomycin or gentamicin).

### 3.3. Postoperative Results

After bone reconstruction, all patients were treated with intravenous antibiotics. The duration of that antibacterial medication, which was changed to oral admission towards discharge from in-patient therapy, varied. Minimum treatment duration was five to seven days and was carried out in two cases (5%). Twenty-one percent of the patients received antibiotics for eight to twenty-one days. Twelve patients (32%) were prescribed antibacterial treatment for three to six weeks, overall. In fifteen patients (39%), the overall duration of antibiotic therapy was more than six weeks.

Follow-up time referring to the last personal consultation of the patient was 11 weeks to 159 weeks, with a mean value of 60 weeks. The standard deviation was calculated to ±36 weeks. In one case, the follow-up consultation was not attended at all.

Concerning the patients’ last consultation, 39% showed complete healing of the bone defect. In twenty cases (53%), radiologic examinations showed partial bone healing in comparison to the status after bone reconstruction. No healing of the bone was found in two patients (5%). One of these patients was treated with surgical revision in May 2024, with decortication of the defect, autologous bone graft from the posterior iliac crest, and addition of bone morphologic protein II. That revision surgery took place nine months after primary reconstruction with Fiberfill©. In November 2024, the patient was seen for consultation with good progress of bone healing.

The other patient was from abroad and did not attend our institute again after a follow-up of 17 weeks. Within that period, no major bone healing could be observed.

In our collective, 78% reached full weight bearing and 19% reached partial weight bearing within our follow-up.

One patient reached no more than partial weight bearing and partial bone healing after a follow-up time of 47 weeks, so that surgical revision with application of bone morphologic protein II was performed in 2024.

In one patient, no weight bearing was accomplished due to a complication with failure of the osteosynthesis (fracture of the plate) and no sufficient healing of the bone within six months. A surgical revision was performed, with decortication of the non-union, autologous bone graft from the posterior iliac crest and attachment of bone morphologic protein II.

Four patients (11%) suffered from re-infection of the osteomyelitis. All these patients were treated with antibiotics and achieved control of the infection again. In only one case, to accomplish infection control, additional surgery was necessary. The re-infections occurred after a mean of 32 days (SD = 18 days).

Overall, there were four acute infections as complications, one mechanical complication with collapse of the osteosynthesis, and two patients with absent/very delayed bone healing.

Complications which could have been associated with Fiberfill© were not observed.

### 3.4. Subgroup Analysis

**Figure 2:**
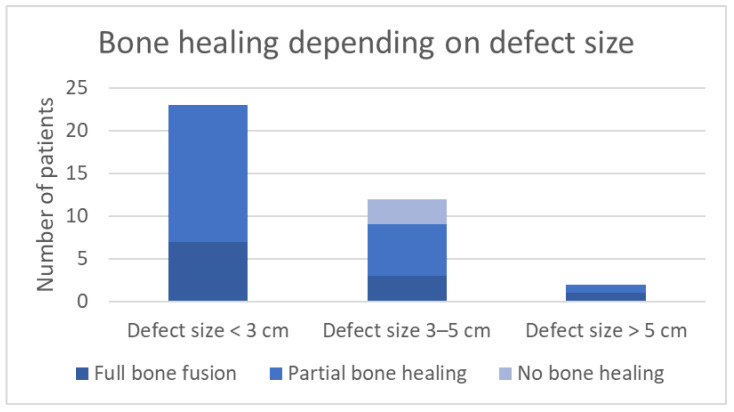
Bone healing depending on defect size.

Concerning bone healing, the results were subdivided into three groups, depending on the size of bone defect (see Figure 2) [21]. Group one contains defect sizes < 3 cm. Group two includes midrange defects of 3–5 cm. Defects measured > 5 cm were included in group three.

**Figure 3:**
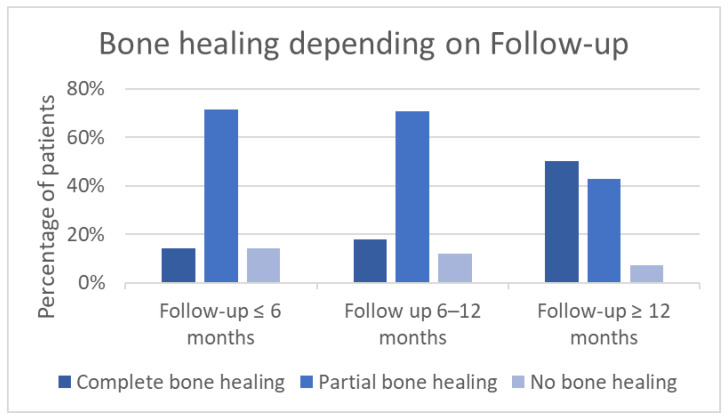
Bone healing depending on time of follow-up.

A subgroup analysis differentiating between follow-up of ≤6 months, follow-up of 6–12 months, and follow-up of >12 months was performed (Figure 3). Isolated observation of patients with a follow-up of >1 year (*n* = 14) in our study demonstrates a complete bone fusion in 50% (total cohort: 39%), while partial bone healing (with progress since last consultation) is found in 43% (total cohort: 53%). There was one non-union after >1 year in this subgroup (7%). In comparison, the subgroup with a follow-up of 6–12 months (*n* = 17) shows a lower percentage of complete bone fusion (18%) and more partial fusion than in longer follow-up. As demonstrated in Figure 3, in the sub-population with a follow-up of ≤6 months the rate of complete bone fusion is inferior to longer follow-up.

As 21 patients (55%) received Fiberfill© as a standalone treatment for bone reconstruction, that group was compared to the 17 patients (45%) who received a combination. Of those patients who received a standalone treatment of Fiberfill©, two were observed with re-infection (11.8%). In the group of combined bone reconstruction with Fiberfill© and autograft/bioactive glass/BMP II, two re-infections occurred (9.5%). Concerning bone healing, complete fusion was achieved in 6 patients (29%) of the solely Fiberfill© group and 5 patients (29%) of the “combination” group. Partial bone fusion was detected in 14 patients (67%) of the “standalone” Fiberfill© group and in 10 patients (59%) of the “combination” group. There was one patient (5%) with no bone healing who received only Fiberfill©, and two patients (12%) who received a combination with Fiberfill©.

**Figure 4:**
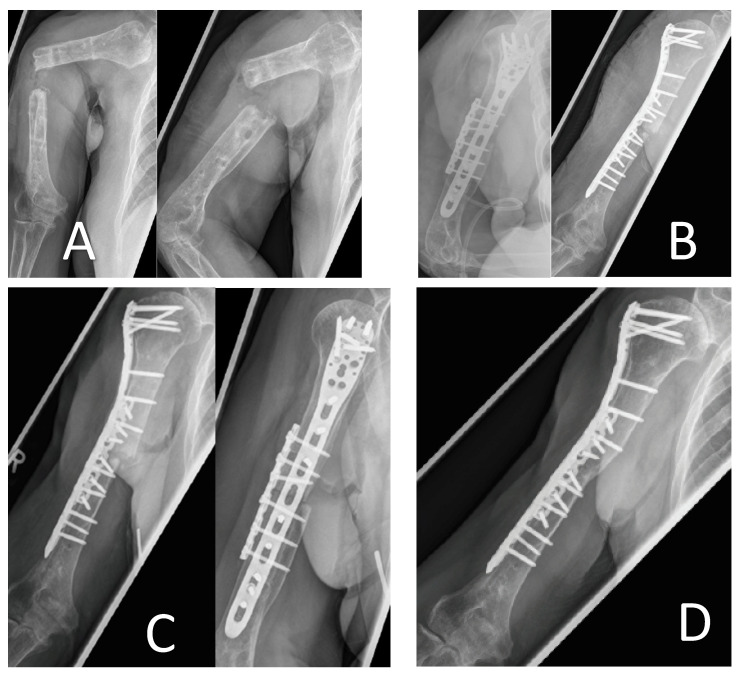
(**A**) Pre-surgery X-rays; (**B**) post-surgery X-rays with plate osteosynthesis and defect reconstruction; (**C**) X-rays at 2 months post-surgery; (**D**) 1.5 years post-surgery X-rays.

The Figure 4 example shows X-ray images of a 70-year-old patient four years after suffering a humeral shaft fracture. After initial nail osteosynthesis, no bone healing occurred. Revision of the osteosynthesis was performed, resulting in a persistent non-union with detection of *Staphylococcus aureus*.

Nail removal and application of an external fixator was performed in March 2022. Removal of the external fixator resulted in an unstable situation, as seen in Figure 4A. In a surgical debridement, *Serratia marcescens* was detected as a pathogen.

Another debridement with additional plate osteosynthesis and minor bone shortening and reconstruction of the 5 mm remaining defect, using 5 ccm Fiberfill©, was performed.

The microbiological probes taken during reconstruction remained without proof of any pathogens. Follow-up after two months showed the beginning of bone healing without secondary dislocation. Ther were no further complications. Complete consolidation was achieved, as shown in Figure 4D.

## 4. Discussion

Therapy of osteomyelitis has been a challenge for generations of surgeons. Two centuries ago, it was Sir Benjamin Brodie who described the changes in bones and joints “blended into a confused mass in the most advanced stage of a disease” [22]. The treatment of these abscesses, named after Sir B. Brodie, has evolved since then.

Fundamental objectives of osteomyelitis therapy remain extensive surgical debridement of bone and soft tissue, and filling of the bone defect. The goals in the therapy of osteomyelitis with bone defects are attained with infection control and bone healing in the months after acute in-hospital treatment.

There are several complications of different severities, which can occur during therapy. Major complications are the recurrence of the infection and delayed or non-healing of the bone. Infection recurrence in COM in the literature is reported to be between 19 and 23% [23,24]. The collective of our study suffered from re-infection in 10.5% of cases. In comparison, the rate of re-infection in our study is rather low. One reason for rather low findings of re-infection could be the limited time of follow-up for some patients. Furthermore, results could be biased, as in severe cases of COM, treating physicians tend to apply established techniques. The gold standard in reconstruction of bone defects in COM remains the autologous bone graft. Therefore, cases anticipated as surpassing the average risk of re-infection or other complications might have been treated rather with autologous spongiosa, than with Fiberfill©.

In our collective, there have been two cases with complications in terms of no bone healing and very delayed bone healing after a follow-up of 47 weeks. Due to the internalization of, e.g., *Staphylococcus aureus* into osteoblasts, and its impairment of the cells, a remaining low-grade infection could be causative here [25,26]. If these patients were considered as infectious-complication cases, re-infection-rate would be calculated as 16%.

More severe complications in untreated or delayed detected osteomyelitis include sepsis, which can be life threatening and cause life-long disablement [11]. None of these severe complications, such as sepsis, were detected.

While the fundamental treatment objectives remained the same during medical progress, osteomyelitis therapy has advanced. Besides research and investigation of antibiotic therapies, new bone substitutes have been developed [7,15]. Between 2008 and 2018, the number of applications of bone substitutes increased by 134% [27]. The global market for bone grafts and substitutes was estimated at a volume of 2.7 billion US Dollars in 2020. By 2028, it is expected to grow to 3.4 billion US Dollars [17].

From antibiotic-loaded acrylic cement to bioactive glass and allogenic bone grafts, various substrates have been developed for bone reconstruction. Many have proven to have reasonable results in recent years, comparable to autologous bone grafts [16,17]. To reduce surgery time and donor-site morbidity, surgeons regularly decide on artificial or allogenic bone substrates, and abstain from autologous bone grafts from iliac crests.

In our cohort, 39% demonstrated a complete bone fusion within time of follow-up. A total of 53% of the collective achieved partial bone fusion. As provided in Figure 3, the performed subgroup analysis demonstrates higher rates of complete bone healing with longer follow-up time. In the literature, rates for complete bone fusion in COM after bone reconstruction with autologous bone grafts are described as being up to 78% [15]. In comparison current litarure investigated bone healing for patients with chronic osteomyelitis who underwent bone reconstruction with a ceramic bone substitute (PerOssal©). Numbers from this study show a bone consolidation in 73% of cases [4]. For other bone substitutes, such as bioactive glass, complete fusion can be observed in 77% of cases [15,28].

Bone healing with complete fusion in 39% and partial healing in 53% of our collective appears to be inferior to the numbers provided in the literature for bioactive glass and autologous grafts. However, time-of-follow-up needs to be considered, which was longer in the cohort of Steinhausen et al., with an average of 31 months for autologous bone graft and 21 months for bioactive glass (14 months’ average in our collective). Armbruster et al. observed their collective during a period of 2 years for their study on ceramic bone substitute. Regarding our collective, the subgroup analysis performed in Figure 4 shows bone healing depending on time of follow-up. In summary, the longer the follow-up period, the higher the rate of complete bone fusion. Simultaneously with the ongoing follow-up, the percentage of partial healing and non-unions decreases. This can be interpreted as ongoing bone healing achieving bone fusion in due course.

In general, due to differences between our cohort and study populations, comparing datasets to each other is always limited. Factors accounting for different results in other studies can be parameters such as time of follow-up, size of the bone defect (see Figure 2), bacteriology, and general health condition.

In our study, 78% of the cohort achieved full weight bearing. That result, in contrast to the 39% with complete radiological bone healing, demonstrates good functioning of the affected limb, which occurred also in cases where no complete bone healing was achieved within the time of follow-up. For the individual patient, weight bearing is certainly the more important outcome, rather than radiographic exams.

Concerning the COM-affected body part, the literature states that more than 90% of chronic bone infections take place in extremities. Occurrence in lower extremities (74%) is observed a lot more often than in upper extremities (18%) [29]. Comparing that data to our findings, where we diagnosed 89.5% of COM in the extremities, our results match the current literature. When comparing upper- vs. lower-limb COM, in our study 74% affected the lower, and 16% the upper, extremities. This distribution indicates an adequate sample, comparable to findings in literature.

Fiberfill© offers the advantage of very high moldability. It can be pressed into the bone defect, to reconstruct it. Depending on the size of the defect, different volumes can be chosen and combination with autologous bone grafts is possible. There is no volume limitation, as it naturally exists in autologous bone grafts; therefore, larger defects can also be addressed. In the field of septic surgery, the long-lasting delivery of antibiotics can be fundamental for infection control. Besides antibiotics, Fiberfill© can be enriched with other liquids (e.g., platelet-rich plasma), when its main use is for rehydration of the Fiberfill© [30].

In comparison to autologous bone grafts, Fiberfill© as an allogenic processed-bone substrate lacks osteogenic properties; it contains no vital cells which could differentiate into bone-forming cells and form ectopic bone [31]. However, it does, presumably, stimulate osteoinduction (osteoblast activation) and osteoconduction. The remains of growth factors after chemical processing of the DBM account for the osteoinduction [17]. As DBM contains far fewer growth factors than bone-morphologic-protein (BMP) substrates, liquid BMP can be added when rehydrating Fiberfill© for retaining a stronger osteoinductive effect. Concerning osteoconduction, the DBM, of which Fiberfill© consists of 75%, serves as a scaffold.

Considering these properties, we did not treat patients with very large defects with solely Fiberfill© but used either a combination of Fiberfill© and autologous graft or autologous graft on its own. Depending on the defect size, combining Fiberfill© and autologous grafts could be an appropriate therapy for larger defects. If difficult to treat bacteria or difficulties to debride surgical sites with cavitary defects are present, antibiotics can be diluted with Fiberfill© and take effect during the following days, with high local concentrations [19]. In our cohort 55% were treated with Fiberfill© as a standalone treatment. Results comparing re-infection and bone fusion with those of the group of a combination therapy with Fiberfill© do not show any disadvantages of the treatment with solely Fiberfill©. Regarding re-infection, results are approximately equal. Concerning bone healing, in this study, Fiberfill as standalone is not inferior to a combination of it with autologous graft/bioactive glass/BMP II, with 29% of complete fusion in both groups. Partial bone healing was observed slightly more often in the “standalone Fiberfill©” group. As mentioned below, a possible bias due to different defect sizes between those two groups has to be considered, though.

The mean defect size before bone reconstruction using Fiberfill© was measured to be 2.4 cm (±1.9 cm SD). In smaller defect sizes, complications such as re-infection and absence/delay of bone healing are observed less frequently. Figure 2 illustrates the fact that within our cohort, in the subgroup of defects < 3 cm, no complication such as “no bone healing” was observed. In comparison, within the group of defect size 3–5 cm, 25% experienced absence of bone healing. Within the third subgroup of defects measuring > 3 cm in size, one patient (50%) showed partial bone healing, while the other could be observed with complete bone healing. Certainly, there is only limited value to these results, especially in subgroup 3, because of the restricted number of included patients. Differences in bone healing regarding defect sizes (</>3 cm), however, comply with our clinical findings. This is also shown by a further subgroup analysis of defect sizes: the mean defect size of reconstructions where solely Fiberfill© was used was calculated to be 1.4 cm (±0.8 cm SD). The mean defect size of treatments with Fiberfill© in combination with autologous grafts, bioactive glass, or BMP II, was 3.8 cm (±2 cm SD).

To our knowledge, there is no literature about treatment of larger defects with Fiberfill©. Following our experience, a combination of Fiberfill© with autologous bone grafts (with osteogenic properties) and BMP (to boost osteoinduction) could achieve reasonable results. The bone substitute could be dissolved in liquid antibiotics, according to prior microbiologic findings, and this would ensure high local antibiotic concentrations with minimum side effects. Treatment of larger defects presumably results in higher rates of complications and re-infections than we found in this study. Possibly, such a modification of the cohort might result in complication rates, e.g., percentage of re-infection, as frequently found in the literature.

### Limitations

Further investigation needs to be carried out to analyze a larger cohort of patients with chronic osteomyelitis and bone reconstruction using Fiberfill©. In our descriptive study design, there is no control group for the patient cohort analyzed, while the collective is heterogeneous regarding different bone and defect types. Some patients do have a follow-up of less than one year. A short follow-up in some patients may underestimate re-infection and other complications. Prospective observation could supply more data, especially referring to long-term infection control and recurrence of chronic osteomyelitis. Also, the course of further bone healing in the group with partial bone-fusion could be observed and analyzed.

## 5. Conclusions

Following the descriptive results of this study, Fiberfill© has the potential to be an appropriate bone substitute for patients with bone defects due to chronic osteomyelitis. In comparison to literature, we did not find higher complication rates in our collective. Regarding larger-size bone defects, according to our observation, a combination of Fiberfill© as an allogenic bone graft with good moldability and availability and an autologous bone graft, can be a considerable treatment option. Further follow-up and studies with a larger collective need to be performed.

## Figures and Tables

**Table 1:** Registered Parameters.

Parameter Group	Parameter	Attributes
Prior to reconstructive surgery	Age	years
	Sex	male, female
	Date of accident	date
	Date of first surgery	date
	Date of infection	date of positive microbiology or histology
	Localization of fracture	humerus, radius, ulna, femur, tibia, fibula, calcaneus, foot
	Type of fracture	closed, open
	Type of fracture	diaphyseal, metaphyseal
	Type of pathogen detected	MSSA, MRSA, coagulase negative *Staphylococci*, *Cutibacteria*, *Enterobacteriaceae*, *Pseudomonadaceae*, fungi, other gram positive, other gram negative
	Defect size	cm
	Number of previous surgeries	<5; 5–10; >10
	Immunosuppression	Yes, No
	Restitution-limiting diseases	diabetes, peripheral artery disease
Index surgery for reconstruction of bone defect	Date of index surgery with Fiberfill©	dated
	Detection of pathogen	Yes, No
	Change of pathogen to prior findings	Yes, No
	Volume of Fiberfill©	in ccm
	Combination of Fiberfill©	Yes, No
	Type of bone supplement for combination	autologous bone graft, allogenic bone graft, bone morphologic protein II, other bone supplement
	Removal of osteosynthesis	Yes, No
	Re-osteosynthesis	Yes, No
	Antibiotic Treatment beginning with reconstructive surgery	Yes, No
	Duration of antibiotic treatment	single shot, up to one week, 2–3 weeks, 3–6 weeks, >6 weeks
Follow-up	Last consultation/Time of Follow-up	in weeks
	Consolidation of defect area	none, partial, complete
	Weight bearing	none, partial, complete
	Re-infection	Yes, No
	Allergies/Side effects after Implant of Fiberfill©	Yes, No
	Osteosyntheses failure	Yes, No
	Revision surgery	Yes, No

## Data Availability

Data presented in this article can be requested from the corresponding authors, due to ethical and privacy restrictions.

## Abbreviations

The following abbreviations are used in this manuscript:

cm	Centimeter
ccm	Cubic centimeter
COM	Chronic osteomyelitis
BMP	Bone morphologic protein
DBM	Demineralized bone matrix/demineralisierte Knochenmatrix
MSSA	Methicillin-sensitive *Staphylococcus aureus*
MRSA	Methicillin-resistant *Staphylococcus aureus*
SD	Standard deviation
ssp.	Species
